# Applying social innovation theory to examine how community co-designed health services develop: using a case study approach and mixed methods

**DOI:** 10.1186/s12913-018-2852-0

**Published:** 2018-01-31

**Authors:** Jane Farmer, Karen Carlisle, Virginia Dickson-Swift, Simon Teasdale, Amanda Kenny, Judy Taylor, Felicity Croker, Karen Marini, Mark Gussy

**Affiliations:** 1 Social Innovation Research Institute, Swinburne University, John Street, Hawthorn, Melbourne, 3122 Australia; 2 College of Medicine & Dentistry, James Cook University, Townsville, QLD 4811 Australia; 3 La Trobe University, Edwards Road, Bendigo, VIC 3550 Australia; 4 Public Policy and Organisations, Glasgow Caledonian University, Cowcaddens Road, Glasgow, G4 0BA Scotland; 5 Rural Nursing, La Trobe Rural Health School, La Trobe University, Edwards Road, Bendigo, VIC 3550 Australia; 6 James Cook University, Townsville, QLD 4811 Australia; 7 College of Medicine & Dentistry, James Cook University, Cairns, QLD Australia; 8 Community Engagement, Murray Primary Health Network, Rowan Street, Bendigo, 3551 Australia; 9 Department of Dentistry and Oral Health, La Trobe Rural Health School, La Trobe University, Edwards Road, Bendigo, VIC 3550 Australia

## Abstract

**Background:**

Citizen participation in health service co-production is increasingly enacted. A reason for engaging community members is to co-design services that are locally-appropriate and harness local assets. To date, much literature examines processes of involving participants, with little consideration of innovative services are designed, how innovations emerge, develop and whether they sustain or diffuse. This paper addresses this gap by examining co-designed initiatives through the lens of social innovation – a conceptualisation more attuned to analysing grassroots innovation than common health services research approaches considering top-down, technical innovations. This paper considers whether social innovation is a useful frame for examining co-designed services.

**Methods:**

Eighty-eight volunteer community-based participants from six rural Australian communities were engaged using the same, tested co-design framework for a 12-month design and then 12-month implementation phase, in 24 workshops (2014–16). Mixed, qualitative data were collected and used to formulate five case studies of community co-designed innovations. A social innovation theory, derived from literature, was applied as an analytical frame to examine co-design cases at 3 stages: innovation growth, development and sustainability/diffusion.

**Results:**

Social innovation theory was found relevant in examining and understanding what occurred at each stage of innovation development. Innovations themselves were all adaptations of existing ideas. They emerged due to local participants combining knowledge from local context, own experiences and exemplars. External facilitation brought resources together. The project provided a protective niche in which pilot innovations developed, but they needed support from managers and/or policymakers to be implemented; and to be compatible with existing health system practices. For innovations to move to sustainability/diffusion required political relationships. Challenging existing practice without these was problematical.

**Conclusions:**

Social innovation provides a useful lens to understand the grassroots innovation process implied in community participation in service co-design. It helps to show problems in co-design processes and highlights the need for strong partnerships and advocacy beyond the immediate community for new ideas to thrive. Regional commissioning organisations are intended to diffuse useful, co-designed service innovations. Efforts are required to develop an innovation system to realise the potential of community involvement in co-design.

## Background

This paper considers how co-designed health services emerge and develop, and their potential for longer-term sustainability and diffusion. It considers the potential for social innovation theory as a frame for understanding what happens. For policy and practice, it highlights that understanding initiatives co-designed with communities as social innovation could help to identify useful strategies in co-design processes so that community co-design may fulfil on its potential to support healthcare change.

The participation of ‘lay’ people (consumers, the public and communities) in aspects of health service production is recommended in policy internationally (Australian Commission on Safety & Quality in Health Care [1, 2]), and increasingly enacted. Lay participation can be in designing and planning services, evaluating, strategizing and governance [3]. One area of health services where lay participation is accepted as significant is in the work of regional commissioning organisations. These exist in countries including England, Canada, New Zealand and Australia [4]. Such organisations bring together service providers, clinicians and local citizens to consider population health data and evidence about what works, to design models of healthcare for local settings [5]. A key driver of community participation – defined as involvement of diverse people that live and/or work locally [6] - is to design and then provide services that align with local context and harness local resources [7, 8]. Regional commissioning organisations aim to identify innovative, efficient, contextually appropriate service models that will improve population health [5]; these might be disseminated across regions, perhaps with adaptation. Here, we investigate the community participation efforts of two Australian regional commissioning organisations – Primary Health Networks (PHNs) – to design and implement locally appropriate service models to improve public oral health.

There is a substantial literature about lay participation in health services, but few studies include multiple cases that allow for comparison. Thinking about the role of communities in producing service innovation, Greenhalgh et al. [9] highlight a gap; published research evidence “focuses on innovations that arise centrally and are disseminated through official channels at the expense of those that arise peripherally and spread informally”. Despite significant interest in lay participation in design, there is a dearth of evidence considering the innovations that community participation might lead to, how these innovations emerge and what happens to them. With an interest in studying community-designed ‘grassroots’ health innovations, we turned to the relatively new notion of social innovation, considering its potential to provide an analytical frame for studying community co-design.

Social innovation as an idea has risen to prominence in relation to movements for citizen involvement in service delivery and novel approaches to welfare. Original usage was sociological, stemming from Gabriel Tarde’s theory that new forms of social relations lead to innovation [10]. Social innovation became widely discussed in the 1990s, with one branch of literature considering innovations developed by civil society actors aimed at creating socio-political change [11]. Within contemporary European policy, social innovations are those that are ‘social in their means and social in their ends’ ([12]; p.35). Brandsen et al. [13] depict social innovation as involving collaborations to co-design and implement solutions to social problems, particularly at local level. According to social innovation theory, co-produced solutions are assumed to have positive societal effects, either through increasing aggregate utilitarian value, or by empowering citizens in innovation processes [14].

Here, our interest lies in the potential of using social innovation ideas and research findings to develop a frame for considering the design, implementation, sustainability and diffusion of co-designed initiatives for providing local health services that come from the ‘grassroots’ of communities. We are interested in how instrumental ‘top-down’ community participation processes (driven by regional commissioners) enable diverse grassroots participants to gather, learn from each other, share knowledge and adapt ideas to new contexts [13, 15]; and then what happens to innovations once they are planned and enacted.

We use data from the Rural ECOH (Engaging Communities in Oral Health) research project that involved community participation in co-design in six rural communities in two Australian states, funded by the Australian National Health and Medical Research Council (2014–16). Discussing our findings as social innovations, we raise questions in this paper, about the development, sustainability and potential for diffusion of initiatives created and consider how this might relate to aspects of process.

## Methods

### Overall project design

The Rural ECOH project aimed to support Primary Health Networks, local service providers and community members to co-design and implement new initiatives intended to improve local oral health; and to study what occurred. At each of six community settings, an evaluated community participation for co-design framework – Remote Service Futures (RSF) [16, 17] - was used to implement a co-design process. RSF centres on four facilitated public workshops covering discussion of: 1) local health and health challenges including using objective health data; 2) evidence-based approaches to local health challenges; 3) exemplar initiatives of services to address health challenges trialled at other communities; 4) and then, co-designing a plan for local health initiatives. Following the co-design process, PHN staff worked with groups of community members and local service providers to implement co-designed ideas. The co-design and implementation processes were facilitated by a university-employed facilitator. Regional commissioning organisations were key drivers/sponsors. In 2014, these organisations were Medicare Locals, but following reconfiguration in 2015, changed to become Primary Health Networks (PHNs) [18]. Mixed-methods data were collected to document what happened during co-design and implementation phases over 24 months. The project had ethical approval from Human Ethics Committees of La Trobe (13–052) and James Cook (H5540) Universities and Queensland Health (13/QTDD/73).

### Community settings included

Six communities were included, with population size ranging from 1150 to 10,400 people and a median of 8670. Community locations were 30–270 min-drive from a large regional hospital (median 150 min-drive). At three settings there were public and private dental practices, while two settings had private dental practices and one township had only visiting dentists. All settings were selected based on health services data showing high incidence of poor dental/oral health compared with state and national averages.

### Co-design process

Community members – defined as people living and working locally - were invited to participate in co-design through: letters sent to local organisations and community groups; advertisements in community newsletters, websites, noticeboards and social media sites; and letterbox drops. Given that, in Rural ECOH, participation was to co-design oral health initiatives, Workshop 1 involved engaging with data about oral health status, while Workshop 2 presented evidence about effectively addressing poor oral health at community level. From this stage, participants identified priority themes for local service innovations. The facilitators brought information about evidence-based projects and initiatives previously undertaken elsewhere, to Workshop 3. This included, e.g. a video about Scottish Government ChildSmile project [http://www.child-smile.org.uk/professionals/about-childsmile.aspx]. Plans featuring the new initiatives wanted by communities, for all settings, were created at Workshop 4. This co-design phase occurred over 12 months.

Eighty-eight community members volunteered to participate across the six settings, through their attendance at one or more of 24 co-design workshops (four at each setting). Numbers attending individual workshops ranged from 3 to 14. At all settings, community members that worked in public services (e.g. health, education and council) participated, and there was also participation of people employed in other sectors, and retirees. Following the design phase, implementation groups were established. Meeting four times over 12 months, their role was to drive the planned changes at each setting to ensure as much action as possible within 12 months, occurred. Implementation group participants included PHN staff, representatives of local service providers and some community members that had also participated in co-design. The lay community members included were those that were interested to stay involved after the co-design process. University-employed facilitators organised the group meetings, took notes and ensured communication between meetings. At two stages, following planning (12 months) and implementation (24 months), cross-community meetings were held in each State, where representatives from all communities met together to discuss their experiences. This was intended to give the participants the experience of being part of a larger research project, to learn from each other and transfer knowledge from one rural community to another. This sharing acted as a motivator and a mechanism for knowledge dissemination between communities across the regions.

### Data collected about co-design and implementation

Formal data collected included: a) typed notes of 24 co-design workshops and 24 implementation group meetings; b) co-designed plans for each setting (six); c) transcripts of audio-recordings of four cross-community meetings (with participants from all settings). In addition, the two facilitators maintained reflective journals covering 2014–16. Informal data were also gathered including notes from informal community conversations and newspaper articles and public social media site postings. Formal written consent was obtained for all recorded outputs. All qualitative data were entered into NVivo for management and analysis.

### Initial data analysis

Following completion of the 12-month implementation phase, Rural ECOH project partners including from universities, PHNs, the Royal Flying Doctors Service and state dental health agencies met for two days to discuss the community co-designed plans, their implementation, project outputs and impacts. It was observed that a set of innovative co-design ideas had been generated - and in several cases actually implemented. We identified cases of innovative initiatives and considered these to be impactful outputs. We decided to examine and explain them.

We took an abductive qualitative approach to data analysis, involving iterations of researcher discussion and working with primary data - and ultimately, comparing with theory [19]. For coding, we followed Richards’ [20] method of descriptive, topic and analytical coding. Three researchers were involved in data analysis, to allow for verification. Eight of the author team were active in various ways in the co-design and implementation processes, so we were able to test and verify our coding and interpretations, through team discussions, with ‘lived experiences’ of the community processes. Exposing our overall data analysis and interpretation to the wider project partner team of health services providers allowed us to further test our interpretations, against their lived experiences of working in and with the communities. Specific steps in this initial analysis are detailed below.

- The project researcher and partner group observed cases of new activities (innovations) at a workshop in April 2016. It was observed that each participating community had developed innovations. There was no community in which there were no innovations.
- Data were initially analysed to identify ‘cases’ of innovations and a set of five were selected for in-depth description and analysis. These were selected from the overall set of innovations designed by communities based on the criteria that they were: a) relatively complex ideas (defined as involving multiple stakeholders and actions) that had arisen from co-design; and b) were seen through to implementation during the 24 months of the Rural ECOH project. Other innovations occurred in each of the six communities during the project, but we judged them as less complex including: information leaflets, provision of free toothbrushes and toothpaste to community members, providing information on oral health in ‘walk-to-school’ programs and an oral health needs assessment. Some ideas were complex, but not delivered during the timespan of the project. These included wanting: fluoridated public water supply; change to a state-provided oral health triage telephone line; and fluoride varnishing for vulnerable children’s teeth.
- Author 1 worked with the two Rural ECOH facilitators to formulate the case study ‘stories’ of innovations, drawing on analysed data.
- Case study drafts were sent to all project researchers and partners for validation or revision.
- Case studies were revised in light of comments (mostly factual corrections around who was involved or precise nature of activities).

### Further data analysis: Comparing case studies with social innovation theory

Having established the case study innovation stories from the analytical process explained above, we sought pre-existing evidence or theory that would help us to examine what had occurred. As noted above, we ultimately drew upon the literature of social innovation. We decided to do this as the innovations we observed were from grassroots and community stakeholder-driven, thus potentially relating more to literature on community-based social innovation, rather than literature about innovation in health services. We did explore the latter, but found it tends to cover the diffusion of top-down and technical health innovations (e.g. [21]).

**Fig. 1:**
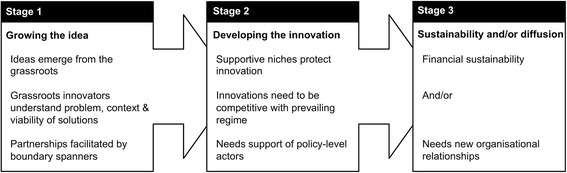
Theory of Grassroots Social Innovation

We applied grassroots social innovation theory to the case studies derived to understand the extent to which the cases might be interpreted as social innovation. We sought to understand if social innovation theory could help in understanding the emergence, implementation, sustainability and diffusion of co-designed ideas about how to provide local health services that come from the grassroots of communities. Based on social innovation literature relevant to that purpose, we derived a model of the grassroots social innovation process highlighting three key stages and significant elements within these (Fig. 1). We applied this model as an analytical frame to the case studies of innovation we had derived from initial analysis. Below, we provide an explanation of the social innovation stages based on literature that we used to inform model development. Literature was derived from a systematic search of Scopus and Google Scholar, identifying literature combining concepts of community and social innovation.

#### Stage 1: Growing the idea

There is an assumption that ideas emergent from system grassroots are significant as they are located close to where problems occur. Thus, innovators understand problems in context and so are more readily able to assess the viability of potential solutions [22]. Social innovation literature suggests that many ideas for community innovations stagnate at the planning stage and are never physically realised [23]. Others suggest innovations can be so contextually enmeshed that their design and implementation necessitates people resourced to negotiate local power structures [24].

This begins to hint at the significance of partnerships between grassroots citizens and policy/service management-level actors if community-led ideas are to flourish beyond the design stage. Grassroots innovation ideas that grow to fruition may be those that coalesce citizens understanding of local problems, context and feasible local solutions; with managers and policymakers understanding of how to navigate new service implementation.

#### Stage 2: Developing the innovation

Grassroots social innovation ideas are fragile and they begin to gain traction only within supportive ‘niches’ ([22], p.1). These protect the new ideas from ‘too harsh selection pressures from incumbent regimes’ ([22], p.2), and nurture them to a piloting stage. Protection and nurturing of new ideas may be particularly significant in healthcare where a conservative technical culture demanding research evidence of cost-effectiveness can stifle survival of new ideas from penetrating the incumbent regime [25]. Again, a supportive political environment may be crucial. People acting as boundary spanners between innovative ideas and regime norms (for example, service managers) could be significant in supporting pilot of new ideas within systems of established practice [26, 27]. Such people may even re-interpret and promote innovations to fit prevailing policy discourses so that they are seen as politically desirable [24, 27].

#### Stage 3: Sustainability and/or diffusion

A novel practice is accepted when it moves to implementation beyond its experimental niche (Jaeger-Erben et al. [28]). Social innovations can be: scaled-up - increased use of the innovation; replicated – used in new locations; and partially translated – where elements of innovations are adopted, adapted and used elsewhere [29]. Raven [30] identified features of innovations that diffuse beyond their niche setting. They involve: a shared vision and expectations among stakeholders; involvement of stakeholders’ social networks for support and resources; and provide opportunities for shared learning among stakeholders. Hatzl et al. [25] explain that shared expectations provide clear direction and feasibility, while shared learning creates feelings of excitement and solidarity between innovators and policy/management actors that can ease innovation acceptance at regime level. Hatzl et al. [25] suggest stakeholder groups involving local policy-level organisations could implement innovation systems to support design, implementation and diffusion of promising community-based initiatives.

To gain acceptance, social innovations often have to negotiate a political as well as practice landscape [31]. Dominant political interests tend to resist change inspired by others [24]. Social innovations tend to diffuse only when compatible with the prevailing macro-level (policy) regime [22]. Given that the more radical social innovation literature highlights the centrality of changing established social and political order [32], the implementation and diffusion of grassroots innovations may require new organizational relationships, including change in operational or governance structures, for example opening-up new roles for ‘lay’/ non-technical community members. It is plausible that where social innovations are commercially profitable such changes might be implemented. For example, Hatzl et al. [25] show how the rise of community renewable energy initiatives has led to community ownership and governance, causing considerable system disruption.

Having devised the three-stage model of grassroots social innovation from literature, we applied it to the case studies of co-designed innovations we derived from Rural ECOH project data. This provided a frame to examine the process of design, development and implementation of innovations, understanding them and the co-design process producing them, as social innovations.

## Results

Below we describe five cases that we identify as relatively complex service innovations that were designed and implemented during the 2014–16 timeline of the Rural ECOH project. We discuss how ideas emerged and developed and the prospects, given current evidence, of their sustainability and diffusion. In the Discussion section, we examine the cases in light of social innovation theory.

### Innovation 1: Health-check reminder stickers

The idea was to develop stickers reminding parents to take their child for a dental check-up. Stickers would be placed into a Child Health Development book by a health practitioner, generally a Maternal and Child Health Nurse (MCHN). Child Health Development books are provided for all children by the Australian government and a system of stickers is used to remind parents of milestones, e.g. immunisation dates. Participants in the co-design process identified that there was no sticker to remind parents about dental checks.

The leaders in identifying this innovation were two community members who had a nursing background, at Settings 2 and 3. As well as noting the idea of the stickers was already established, they said the idea because was equitable; stickers would stimulate a conversation with all parents and health practitioners would not be seen as ‘picking on’ individuals. PHN staff supported the idea by having stickers designed and produced, with a printable template placed on the PHN website for others to use. The stickers were used during the Rural ECOH project, but wider diffusion requires a sustained promotional campaign beyond the project.

### Innovation 2: ‘Dry’ toothbrushing program

This innovation was developed for use at schools that do not have access to hygienic sinks. Community participants in co-design at Setting 2 included local school and dental clinic staff. Having seen examples of toothbrushing programs at Workshop 3, community participants asked the project facilitator for help to research programs that could be implemented at a local school where there was no access to clean sinks. This resulted in designing a school-based ‘dry’ toothbrushing program plus new dry tooth-brushing guidelines for schools. The guidelines and innovation were then tested in a pilot project by Dental Health Services Victoria (State government dental agency).

### Innovation 3: Classroom toothbrush holder

The innovation is a cheap, hygienic toothbrush rack to hold the toothbrushes of a class of school students. Hygienic storage is important for school-based toothbrushing programs (i.e so brushes do not touch each other); and mobility is important so brushes can be moved for cleaning. Existing ‘brush-bus’ products were explored, but found to be expensive and small-size. Participants at Setting 2 designed a cheap, hygienic, portable, large brush-container.

Three different-sized plastic containers and a new drill ‘bit’ were purchased. Holes were drilled in the ‘brush-buckets’ at spaces so that brush-heads would not touch; and different sized containers arranged so that brushes were kept separate and sealed by a large lid, for clean transportation plus drainage and air-drying (important for infection control). The total cost was $14, compared with around $100 for existing ‘brush-bus’ products. The new container was used successfully in Setting 2. Overall, the main challenge to sustaining the toothbrushing program was identifying volunteers to assist at schools.

### Innovation 4: Oral health education and screening at school immunisation sessions

This innovation involves using time in school immunisation sessions to provide oral health screening and/or education. The idea was suggested by community members that worked as school nurses and teachers. They identified time while school students are waiting before and immediately after immunisations, to provide other health inputs. The idea was that teachers or school nurses - appropriately trained - could provide oral health education and/or screening. Later the idea of deploying dental students on local placements, emerged as another resource.

An initial challenge was discovering who should ‘approve’ this innovation – stakeholders included the area school immunisation co-ordinator, directors of oral health services and school principals; all of whom were engaged by the Rural ECOH facilitator through contacts with the PHN as lead agency for the project.

Engaging the university dental students involved agreement from university placement co-ordinators. Ultimately, this involvement of dental students emerged as having potential to be sustainable. Involving the students harnessed an ‘additional’ resource which avoids negotiating extra work for existing health and school staff, although they do still have to co-ordinate the sessions. Existing practitioner working circumstances are not affected as student inputs are episodic and for learning purposes. The students suggested they enjoyed helping out and they gained experience of working in a school. The university can help to sustain the initiative by sending students who are on clinical placements locally. However, if ongoing provision of the service innovation hinges on student inputs, then the initiative relies on maintaining enthusiasm of university staff and finding resources for student travel.

### Innovation 5: Oral health training program for non-oral health practitioners

This innovation is a one-day face-to-face training program in oral health to equip local individuals who are not oral health specialists, to conduct oral health education and basic screening (e.g. at school immunisation sessions). The idea arose because community participants at Setting 4 saw the example of the “Lift the Lip” screening program presented at Workshop 3.

An existing Australian training program was identified, but it was deemed overly expensive so the Rural ECOH facilitator commenced development of a bespoke course for local participants. She had a experience of educational design and garnered other input from staff of local service providers and state dental agencies, some of which were Rural ECOH partner organisations. The facilitator drove development because health agencies were reorganising at that time and she was tasked with ensuring the initiative happened. Funding to develop and run the training program was provided by a Medicare Local. A Registered Training Organisation (RTO) provided formal assessment so participants would receive a qualification from an accredited provider. Ultimately, 17 participants were trained.

Following training, at Setting 4, discussions in health and education organisations led to a decision that only formal oral health practitioners should conduct screening. No reasons were formally given for this decision. There were informal suggestions it could be linked to concerns about appropriate use of staff time and/or to concerns over practitioners’ scope of practice. At Setting 5, where younger children were the focus, MCHNs received training and went on to conduct oral health checks at pre-school. This new practice may be more straightforward to implement in this setting because it aligns with existing child health practices and parents are present to give consent.

Over the longer term, the RTO and the Medicare Local that funded training program development, both disbanded. This threatens the program’s longer-term sustainability, although some local organisations have expressed an interest in hosting it.

## Discussion

In Rural ECOH, community participation led to co-design of a range of innovations, including some quite complex innovations highlighted here that involved multiple stakeholders and actions that were implemented during the 24-month project timeline. As indicated in explanation of the cases above, and summarised in Table 1, some of these innovations may be unsustainable in the longer term. Below, we use grassroots social innovation theory as a frame to examine the community-based ideas that emerged, development of innovations and to consider longer-term impacts. Based on our use, we suggest that grassroots social innovation theory is useful for analysing how to improve aspects of community participation for co-designed services.

**Table 1:** Fit with Grassroots Social Innovation Theory

	Stickers	Dry tooth-brushing	Brush-containers	Education / screening at immunisation	Training program
Growing the idea – innovations all involved adapting existing product/service concepts					
*Idea is from grassrooots*	√	√	√	√	√
*Local innovators understand problem & produce feasible solutions*	Adapted based on existing sticker system	Adapted based on lack of access to hygienic sinks	Adapted to produce cheaper product that met needs	Adapted for practice ‘space’ opportunity seen	Adopted, based on programs elsewhere
*Partnership facilitated by boundary spanning people*	PHN staff supported idea to print stickers	Local participants examine evidence with researcher	Project stimulates school staff to produce new container	School staff linked with immunisation co-ordinators by project	Facilitator harnesses Medicare Local & RTO^a^ to provide
Development – all innovations were nurtured into implementation in the project ‘niche’					
*Niche protection*	√	√	√	√	√
*Making it compatible with existing regime/practice*	Fits with national child development book	Produce evidence-based guidelines	Fit with guidelines. Met hygiene standards	Sought approval of schools, PHNs^b^ & other agencies	Appeared compatible with health system at first
*Support of policy level actors*	PHN printed stickers and put on website	Adopted by State dental agency		Supported by PHNs, School Management & University.	Affected professional boundaries
Sustainability and diffusion – some innovations may not continue					
*Do we think it will continue?*	~maybe	√	~	√	~
*New organizational relationships*	Needs PHN promotion	State agency diffused across state	Local innovation	Between schools and University	At first, but challenges professional boundaries
*Financial sustainability*	Low cost, but uncertain	Depends on schools that implement it	Remains a local innovation	Dependent on dental student input	Seeking host agency for program

^a^*RTO* Recognised Training Organisation

^b^*PHN* Primary Health Network

In constructing the three-stage model of grassroots social innovation we showed that the process of social innovation from idea to diffusion requires a series of careful balancing acts. Ideas - adapted for local context - can be formulated within communities by gathering an appropriate assemblage of citizens and knowledge. An example is Case 2 where the facilitator provided a literature search to assist community members with developing a toothbrushing guideline. Implementation of ideas requires a protected niche. In our study, the Rural ECOH project provided funding, human resources and interest of PHN managers so experimentation could occur in relatively unthreatened circumstances. For the subsequent innovations to be implemented and diffused, the support of management and policy level actors appears necessary. In our study, this was borne out by the rapid implementation and diffusion of guidelines in Case 2 when the state dental health agency provided support, while Case 5 has an uncertain future because service managers became dubious about non-oral health practitioners undertaking training.

### Growing the idea

Unsurprisingly given the methodological approach of Rural ECOH, the ideas emergent from co-design tended to be inspired by initiatives previously developed and tried elsewhere (we showed these at co-design Workshop 3). The innovations developed form a loose typology of those: extending an existing successful practice (stickers); developing a cheaper version of an existing product (toothbrush container); adapting an existing practice for a different context (dry tooth-brushing); applying an existing practice in a new practice ‘space’ (adding oral health education and screening into an immunisation program); and, directly translating an idea from existing evidence (oral health training program).

Given our placing of co-design as social innovation (as a process itself, but also producing social innovations as outputs), it is significant to note how adaptation of existing evidence and initiatives for local contexts, occurred. Firstly, there were contributions of community participants who were also services practitioners (council, education, health). All of the cases had public services workers central to the initial idea and/or its implementation – child health nurses (Case 1), teachers and oral health clinic staff (2 and 3), school nurses and teachers (4) and multiple health practitioners, managers and educators (5). In creating feasible solutions to local challenges, they combined their knowledge of: a public health challenge, local context and how health systems function. The well acknowledged phenomenon of boundary crossers applied in these rural communities [33]. Health and other public service professionals were service deliverers but also were community members. Such people can contribute from their own lived experience of the community but also from their professional experience. Facilitators tended to act as bricoleurs [34]. They assembled helpful resources, including: networks of local people with useful knowledge; and external actors including public sector managers, policy-level stakeholders (such as state dental health agency) and university staff. For example, in Case 5, the facilitator brought together health professionals and managers with ideas, university educators to design a program, a RTO to accredit training and funding from a Medicare Local. As well as connecting community participants and other resources, the facilitators - because they worked at the behest of policy-level organisations, but also at arms-length distance from senior managers - were able to provide an empowering space where community participants were both ‘given permission’ to consider service change and time-out from everyday routines, to draw on their knowledge and be creative. While this protective niche was significant for growing innovations, having facilitators to act as bricoleurs was an added resource brought about using project monies. The artificial nature of this resource raises the question of whether innovation-generating opportunities could be made more widespread - because that would require considerable resources.

In disseminating evidence-based ideas from literature and other communities, Rural ECOH’s facilitators informed and empowered community discussions and this resulted in generating new initiatives. This shows that, effectively, co-design projects can themselves act as a means of diffusing innovation and disseminating ideas between settings.

### Development of innovations

The Rural ECOH project provided a supportive niche for innovations to be designed, developed and protected. Working with PHN staff to implement the initiatives, facilitators’ roles as bricoleurs was also significant as ideas were developed and ‘nurtured’. They acted to connect emerging innovations with policy-level and management actors, bringing initiatives to the attention of those who could harness resources and aligning them with system practices. For example, when participants at Setting 2 decided to create a dry toothbrushing program, the facilitator linked them with State dental health agency staff. Quite quickly, this State agency obtained funding to trial dry toothbrushing in other places. Draft guidelines that were drawn-up by the facilitator with community members, were refined, endorsed and used by the State agency.

Case study 5 exemplifies where implementation apparently faltered, perhaps because the innovation was insufficiently embedded within the existing health regime. For reasons that were valid given health system disruption at the time, the facilitator developed materials of the oral health training course herself. Once the initial supporting organisations had disbanded, there was insufficient buy-in from other partners that affected the sustainability of running training. As well, discussion around scope of practice and burdens of additional work, affected who was ‘allowed’ to undertake oral health screening in the communities. It is arguable that, in this case, partnerships with stakeholders were insufficiently developed to ease the path to uptake of new practice. Particularly, forging strong partnerships with policy level actors representing affected health practitioner groups might have helped.

Where innovations were successfully implemented they tended to be presented as compatible with existing regimes. For example, the stickers in case study 1 were an extension of established practice.

It might be argued that the range of initiatives designed by communities appear relatively conservative – perhaps particularly if compared with literature where communities develop sophisticated initiatives such as renewable energy schemes [23]. We can speculate on reasons why this might be the case. First, many community member participants tended to have little initial knowledge about rural oral health deficits and so the participatory process was partly an exercise in awareness-raising. Second, PHNs sponsored the process, thus the range of potential activities had to fit within the PHN remit and scope for action – i.e. largely about public health improvement and with little direct power to change clinical dental services. Third, participants tended to be influenced by the group of exemplar initiatives brought at Workshop 3 (although the choice of these was guided by their interests). Fourth, there was an implicit understanding that community members would be involved in implementing the initiatives and that there would be minimal resources, as the PHN did not have new resources. Finally, we only scrutinised here, ideas of communities that were fully implemented during the 24 months of Rural ECOH. Community members did raise other ideas that would take longer to deliver; for example, water fluoridation was desired at one setting. We do not judge that community co-design will always be conservative, but rather it is constrained by contextual factors and, understanding these, community members tend to be shrewd about expending their efforts. Desiring fluoridation of the water supply was a suggested innovation and is not a conservative activity. While it, and other less conservative innovations that were suggested, did not happen in 24 months, community members may have built capacity and networks that they can apply - after Rural ECOH - to pursue their more adventurous aspirations into the future.

### Sustainability and/or diffusion

The Rural ECOH project was completed recently so insufficient time has passed to evaluate which, if any, of the implemented innovations will persist. It appears that all are technically feasible, given their derivation from exemplars and existing practices. Consideration of social innovation theory gives us avenues to predict what could occur. Projected cost savings may be of minor importance to sustainability. Two innovations (Case studies 3 and 5) that demonstrate potential cost savings may not be sustainable, or diffuse. Case Study 3, produced a low-cost toothbrush holder, but so far as we know, the design has not been taken-up elsewhere. And the toothbrushing program itself was threatened, in the setting, due to lack of local volunteers. It is also noteworthy that Rural ECOH innovations were generated within a healthcare regime focused on operational service provision and without a systemic mechanism for identifying and promoting innovation diffusion between sites. The Primary Health Networks have interest in identifying and diffusing promising innovation, but there is little guidance, as yet, of how this might occur (in policy or research literature).

Implementation and diffusion seems particularly dependent on harnessing policy-level support to demonstrate the compatibility of innovations with prevailing system/regimes. This point is exemplified by Case 5 where the innovation of a training program failed to gain a new owner at policy level when its original sponsors disbanded, threatening its long-term viability.

One case demonstrates successful diffusion outside the immediate setting. Case study 2 is a novel dry toothbrushing program (developed from grassroots, adapted from an exemplar) that has been adopted by Dental Health Services Victoria. Key to this we suggest, is that policy-level support and funding for development, were successfully and quickly engaged. The innovation has thus been accepted within the existing health regime. Together these findings imply that if grassroots health innovations are to be diffused / achieve sustainability, then they need to be presented as compatible with the existing regime, and that innovators need to actively seek support from policy and management level actors.

It is significant to remember the suggestion that social innovations are not all readily transferrable between contexts. Larsson and Brandsen [24] say that many social innovations are so context-specific that it is unrealistic to expect them to succeed elsewhere. This may pertain to the grassroots health innovations we have considered, although superficially all appear to be ideas that could be widely applicable.

## Conclusions

Our study provides useful findings for policy and practice. Firstly, community members will design and participate in implementation of innovative health activities over a prolonged period of time. This could be expedited with the support of a skilled facilitator to pull together diverse people and information. However, this would be costly if community participation in co-design and implementation was to be scaled up. Drawing on the terminology of social innovation – protective niches are expensive to resource at scale. This leads into our second suggested useful finding which is that literature of community social innovation appears insightful in informing about processes for bringing the innovative ideas from community co-design into fruition as service innovations. We think, when working with communities, that evidence from other fields about how communities implement activities, e.g. community energy initiatives, could be more informative than the literature of health service innovation which tends to examine top-down organisation and health system innovation. Thirdly, we found that – while driven to involve communities in designing contextually appropriate services - PHNs and local service providers were actually unused to dealing with accommodating these ideas into practice. If community co-design is to occur more widely – and indeed to change the health system to be more customer-centric and community governed, then understanding how systems can accommodate novel ideas from the community, requires greater consideration in health policy. We suggest that insufficient thought has been given to potential outcomes of involving diverse partners in service design – i.e. that innovations will emerge, require to be accommodated within a conservative system and might be usefully transferred across communities, at least in part. This causes us to warn – be careful what you wish for, in the health system, when you invite community members to design innovative services. The situation at least calls for regional commissioning organisations to consider mechanisms for disseminating fruitful service innovations within their jurisdictions. In social innovation theory terms, most of the effort to date seems to be on ‘growing the idea’ in community participation – with less thought given to development, sustainability and diffusion phases.

Considering more theoretical contributions of our work, we propose that deployment of social innovation as an analytical frame for analysing community coproduced healthcare initiatives is useful. It addresses the apparent gap in literature covering the implementation of community co-designed innovation.

Through the lens of social innovation theory, we identified that community participation in co-design led to low-cost and technically feasible evidence-based service and product innovations. These were grounded in local community members’ experiences of problems and context. The key facilitators of effective co-design were identified as i) protective niche to generate ideas; ii) local compatibility to implement ideas and iii) political relationships to sustain changes.

The Rural ECOH community participation in co-design project created a supportive niche for innovations to be tried. Our findings highlight that low costs and technical feasibility are not in themselves sufficient for grassroots innovations to be sustained or diffused. Social innovation theory highlights the need to engage stakeholders at meso (management) and macro (policy) levels of systems to diffuse innovations. At the micro (community organisation) level this means that innovators / bricoleurs need to craft innovations as consistent with existing regimes and engage powerful stakeholders, leading to innovation take-up and advocacy.

The concept of social innovation (as understood in public policy) is built on suggestions that reframing social networks enables less powerful actors with a greater understanding of social problems to co-design new solutions. Our data suggest that - applied to the health context - this holds true. Such a system requires an acceptance that with innovation comes risk, but community participants can start with relatively non-disruptive (or conservative) changes, as participants in Rural ECOH communities did. However, this would appear to rule out locally-generated ideas likely to challenge existing funding and policy structures. The case studies generated relatively safe ideas such as reminders, toothbrush racks and education, raising the question as to whether conservatism is an inherent limitation of co-design processes that are actually driven instrumentally by health organisations? Understanding what to do with grassroots innovation when it occurs seems an essential next step if consumer and community partnership is to grow and fulfil on its promise. Otherwise community stakeholders might become disillusioned when managers and policymakers do not implement their power to trial, but also to accommodate innovations that address local problems through harnessing local resources.

## Abbreviations

ACSQHC: Australian Commission on Safety & Quality in Health Care
ECOH: Engaging Communities in Oral Health
MCHN: Maternal and Child Health Nurse
PHN: Primary Health Network
RSF: Remote Service Futures
RTO: Registered Training Organisation
